# Supplementary material for: Hijacking of multiple phospholipid biosynthetic pathways and induction of membrane biogenesis by a picornaviral 3CD protein
Source: PLoS Pathog. 2018 May 21;14(5):e1007086. doi: 10.1371/journal.ppat.1007086 (PMC5983871; doi:10.1371/journal.ppat.1007086)
Supplement: S1 Fig — (A) HeLa cells were infected with WT PV at an MOI of 10. A lysate was prepared 4 h post-infection and used for an GST-GGA3-pull-down assay as outlined in Fig 3A. with subsequent Western blotting and detection using anti-Arf1 antibody. Arf1-GTP (bead-eluted material) and total Arf1 (from unfractionated lysate) were detected by Western blot using the indicated antibodies and enhanced chemifluorescence. (B) The magnitude of Arf1 activation was determined by quantifying the fluorescence intensity of Arf1-GTP and total Arf1 and reporting the quotient thereof. The averages of the values for the quotients (n = 4) were: 1.27 ± 0.14 (SEM) in mock-infected cells and 8.37 ± 0.03 (SEM) in WT PV-infected cells. The difference was significant based on a Student’s t-test, which gave a P value of 0.0001. (PDF) [file ppat.1007086.s001.pdf]

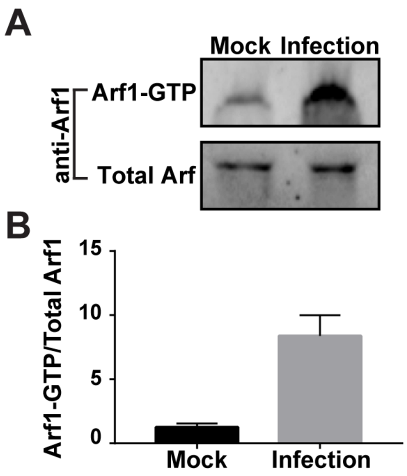

S1 Fig. **Poliovirus infection causes activation of Arf1. (A)** HeLa cells were infected with WT PV at an MOI of 10. A lysate was prepared 4 h post-infection and used for an GST-GGA3-pull-down assay as outlined in **Fig. 3A**. with subsequent Western blotting and detection using anti-Arf1 antibody. Arf1-GTP (bead-eluted material) and total Arf1 (from unfractionated lysate) were detected by Western blot using the indicated antibodies and enhanced chemifluorescence. **(B)** The magnitude of Arf1 activation was determined by quantifying the fluorescence intensity of Arf1-GTP and total Arf1 and reporting the quotient thereof. The averages of the values for the quotients (n=4) were: 1.27 ± 0.14 (SEM) in mock-infected cells and 8.37 ± 0.03 (SEM) in WT PV-infected cells. The difference was significant based on a Student's t-test, which gave a P value of 0.0001.
