# Supplementary material for: Hijacking of multiple phospholipid biosynthetic pathways and induction of membrane biogenesis by a picornaviral 3CD protein
Source: PLoS Pathog. 2018 May 21;14(5):e1007086. doi: 10.1371/journal.ppat.1007086 (PMC5983871; doi:10.1371/journal.ppat.1007086)
Supplement: S2 Fig — (A) HeLa cells were infected with PV at an MOI of 10. Four hours post-infection, cells were immunostained for the following: (i) 3D/3CD (ii) Arf1, (iii) GBF1, or (iv) PI4KB (green). Infected cells were identified by changes to pattern of PI4P expression (red). The nucleus was stained with DAPI (blue). (B) Experiment performed as above immunostained for the following: (i) Arf1, (ii) GBF1, or (iii) PI4KB (green). Infected cells were identified by 3D/3CD expression (red). The nucleus was stained with DAPI (blue). (C) Experiment performed as above immunostained for the following: (i) Giantin or (ii) Calnexin (green). Infected cells were identified by 3D/3CD expression (red). The nucleus was stained with DAPI (blue). (PDF) [file ppat.1007086.s002.pdf]

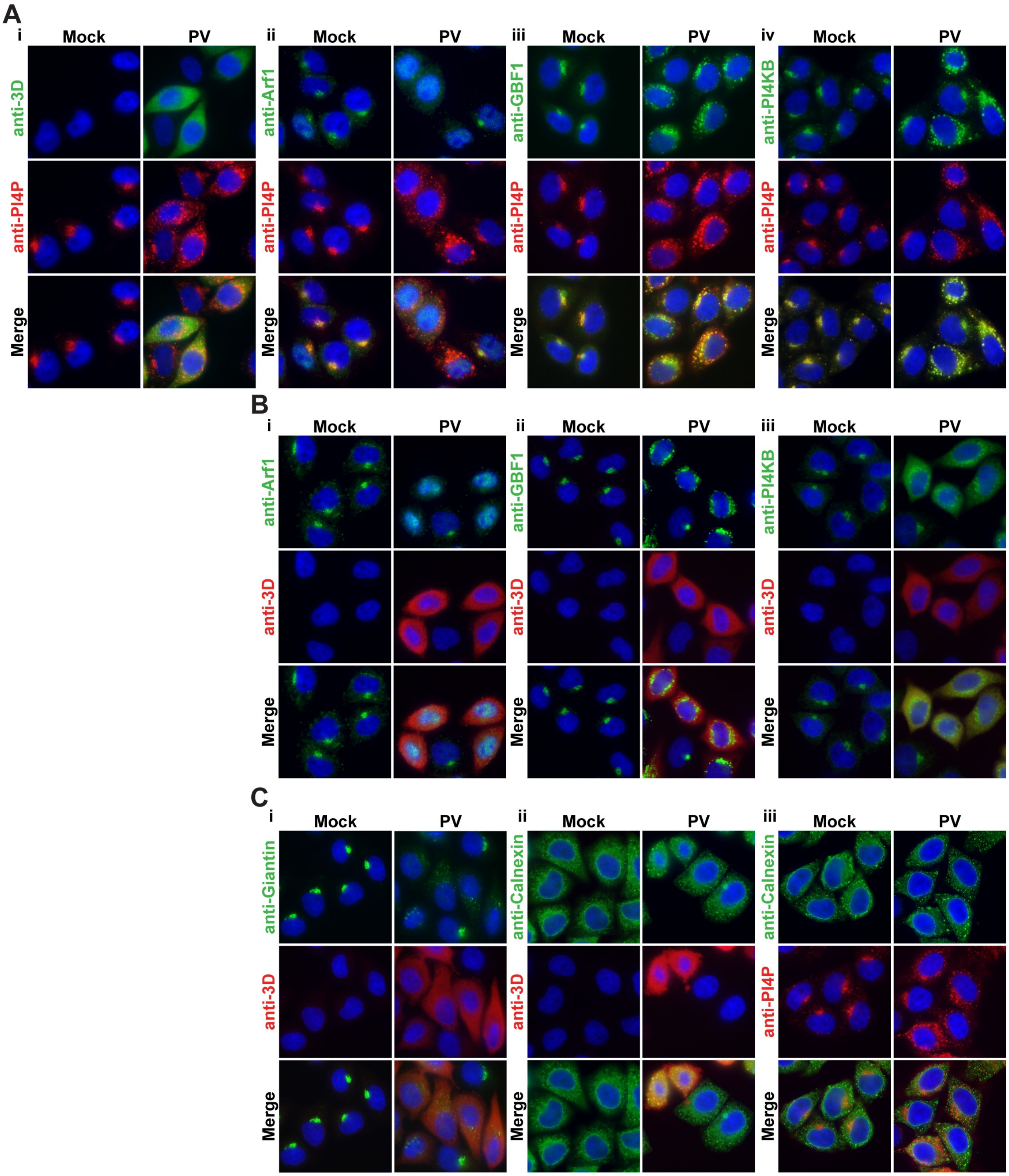

S2 Fig. **Changes to Arf1, GBF1, and PI4KB caused by 3CD are also observed during PV infection. (A)** HeLa cells were infected with PV at an MOI of 10. Four hours post-infection, cells were immunostained for the following: **(i)** 3D/3CD **(ii)** Arf1, **(iii)** GBF1, or **(iv)** PI4KB (green). Infected cells were identified by changes to pattern of

PI4P expression (red). The nucleus was stained with DAPI (blue). **(B)** Experiment performed as above immunostained for the following: **(i)** Arf1, **(ii)** GBF1, or **(iii)** PI4KB (green). Infected cells were identified by 3D/3CD expression (red). The nucleus was stained with DAPI (blue). **(C)** Experiment performed as above immunostained for the following: **(i)** Giantin or **(ii)** Calnexin (green). Infected cells were identified by 3D/3CD expression (red). The nucleus was stained with DAPI (blue).
