# Supplementary material for: Hijacking of multiple phospholipid biosynthetic pathways and induction of membrane biogenesis by a picornaviral 3CD protein
Source: PLoS Pathog. 2018 May 21;14(5):e1007086. doi: 10.1371/journal.ppat.1007086 (PMC5983871; doi:10.1371/journal.ppat.1007086)
Supplement: S3 Fig — (A) The indicated 3CD derivatives were expressed individually in HeLa cells and immunostained for the presence of PI4P (red) and 3CD (green). The nucleus was stained with DAPI (blue). Neither 3CD derivative altered the level of PI4P or its localization. (B) Quantification of PI4P intensity per cell (n = 20) was performed as described in the legend to Fig 1C. The averages of the normalized values were: 1.06 ± 0.02 (SEM) in mock-transfected cells; 1.09 ± 0.06 (SEM) in 3CR13LD-transfected cells; 0.95 ± 0.05 (SEM) in 3CR84LD -transfected cells. The level of PI4P induction observed in 3CD mutant-transfected cells was not significant when compared to mock-transfected cells based on a Student’s t-test. (C) Activation of Arf1 by the 3CD derivatives was determined as described in the legend to Fig 3A. Both derivatives induced activation of Arf1. (D) The magnitude of Arf1 activation was determined as described in the legend to Fig 3B. The averages of the values for the quotients (n = 3) were: 0.43 ± 0.03 (SEM) in mock-transfected cells; 1.23 ± 0.18 (SEM) in 3CR13LD-transfected cells; 1.50 ± 0.15 (SEM) in 3CR84LD-transfected cells. For mock vs 3CR13LD, mock vs 3CR84LD and 3CR13LD vs 3CR84LD, a Student’s t-test yielded P values of 0.0112, 0.0024 and 0.3169, respectively. (E) Complementation of 3CR13LD- or 3CR84LD-expressing subgenomic replicons by 3CD-GAA. HeLa cells were transfected with replicon RNA indicated in the key. GAA refers to a replicon expressing a catalytically inactive 3D-encoded polymerase; the corresponding 3CD-GAA should function normally in PI4P induction. Luciferase activity was measured every hour post-transfection as indicated. (PDF) [file ppat.1007086.s003.pdf]

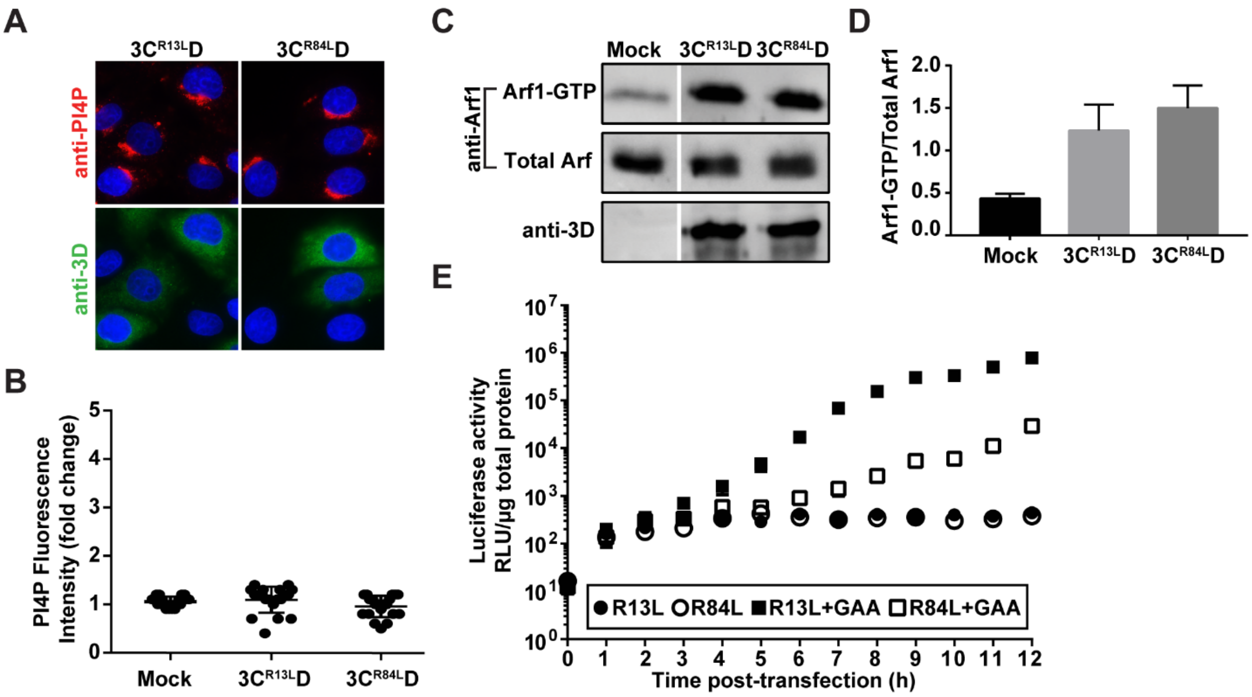

S3 Fig. **Additional 3CD variants with substitutions in the 3C domain exhibit defects to PI4P induction caused by a block at a step post-Arf1 activation. (A)** The indicated 3CD derivatives were expressed individually in HeLa cells and immunostained for the presence of PI4P (red) and 3CD (green). The nucleus was stained with DAPI (blue). Neither 3CD derivative altered the level of PI4P or its localization. **(B)** Quantification of PI4P intensity per cell (n=20) was performed as described in the legend to **Fig. 1C**. The averages of the normalized values were: 1.06 ± 0.02 (SEM) in mock-transfected cells; 1.09 ± 0.06 (SEM) in $3C^{R13L}D$-transfected cells; 0.95 ± 0.05 (SEM) in $3C^{R84L}D$ -transfected cells. The level of PI4P induction observed in 3CD mutant-transfected cells was not significant when compared to mock-transfected cells based on a Student's t-test. **(C)** Activation of Arf1 by the 3CD derivatives was determined as described in the legend to **Fig. 3A**. Both derivatives induced activation of Arf1. **(D)** The magnitude of Arf1 activation was determined as described in the legend to **Fig. 3B**. The averages of the values for the quotients (n=3) were: 0.43 ± 0.03 (SEM) in mock-transfected cells; 1.23 ± 0.18 (SEM) in $3C^{R13L}D$-transfected cells; 1.50 ± 0.15 (SEM) in $3C^{R84L}D$-transfected cells. For mock vs $3C^{R13L}D$, mock vs $3C^{R84L}D$ and $3C^{R13L}D$ vs $3C^{R84L}D$, a Student's t-test yielded P values of 0.0112, 0.0024 and 0.3169, respectively. **(E)** Complementation of $3C^{R13L}D$- or $3C^{R84L}D$-expressing subgenomic

replicons by 3CD-GAA. HeLa cells were transfected with replicon RNA indicated in the key. GAA refers to a replicon expressing a catalytically inactive 3D-encoded polymerase; the corresponding 3CD-GAA should function normally in PI4P induction. Luciferase activity was measured every hour post-transfection as indicated.
