# Supplementary material for: Hijacking of multiple phospholipid biosynthetic pathways and induction of membrane biogenesis by a picornaviral 3CD protein
Source: PLoS Pathog. 2018 May 21;14(5):e1007086. doi: 10.1371/journal.ppat.1007086 (PMC5983871; doi:10.1371/journal.ppat.1007086)
Supplement: S4 Fig — PIP2 is located at the plasma membrane and can only be detected in naïve HeLa cells at 0°C [51]. We confirm the activity and specificity of the antibody used in this study by immunostaining for PIP2 (red) at 0°C. The nucleus was stained with DAPI (blue). (PDF) [file ppat.1007086.s004.pdf]

A

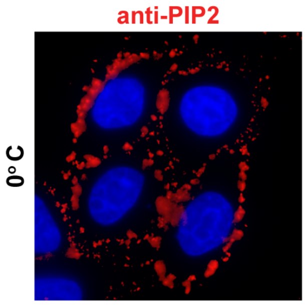


S4 Fig. **Specificity of the anti-PIP2 antibody.** PIP2 is located at the plasma membrane and can only be detected in naïve HeLa cells at 0 $^{o}$C [51]. We confirm the activity and specificity of the antibody used in this study by immunostaining for PIP2 (red) at 0 $^{o}$C. The nucleus was stained with DAPI (blue).
