# Supplementary material for: Hijacking of multiple phospholipid biosynthetic pathways and induction of membrane biogenesis by a picornaviral 3CD protein
Source: PLoS Pathog. 2018 May 21;14(5):e1007086. doi: 10.1371/journal.ppat.1007086 (PMC5983871; doi:10.1371/journal.ppat.1007086)
Supplement: S5 Fig — Effect of actinomycin D (AMD) on 3CD-mediated induction of PIP2 (A) and PC (B). HeLa cells were maintained in the absence or presence of AMD (5 μg/mL) prior to transfection with 3CD mRNA. Cells were immunostained for PIP2 or PC (red) and 3CD (green); the nucleus was stained with DAPI (blue). The presence of AMD did not interfere with induction of PIP2 or PC. (C) The indicated 3CD derivatives were expressed individually in HeLa cells and immunostained for the presence of PIP2 (red) and 3CD (green). The nucleus was stained with DAPI (blue). Neither 3CD derivative impaired induction of PIP2. Quantification of PIP2 intensity per cell (n = 30) was performed as described in the legend to Fig 1C. The averages of the normalized values were: 1.03 ± 0.07 (SEM) in mock-transfected cells; 4.71 ± 0.27 (SEM) in 3CR13LD-transfected cells; 4.08 ± 0.27 (SEM) in 3CR84LD-transfected cells. The level of PIP2 induction observed in 3CD mutant-transfected cells was not significantly different when compared to WT 3CD-transfected cells based on a Student’s t-test. (D) The indicated 3CD derivatives were expressed individually in HeLa cells and immunostained for the presence of PC (red) and 3CD (green). The nucleus was stained with DAPI (blue). Neither 3CD derivative impaired induction of PC. Quantification of PC intensity per cell (n = 20) was performed as described in the legend to Fig 1C. The averages of the normalized values were: 1.00 ± 0.04 (SEM) in mock-transfected cells; 2.52 ± 0.14 (SEM) in 3CR13LD-transfected cells; 2.02 ± 0.11 (SEM) in 3CR84LD-transfected cells. The level of PC induction observed in 3CD mutant-transfected cells was not significantly different when compared to WT 3CD-transfected cells based on a Student’s t-test. (PDF) [file ppat.1007086.s005.pdf]

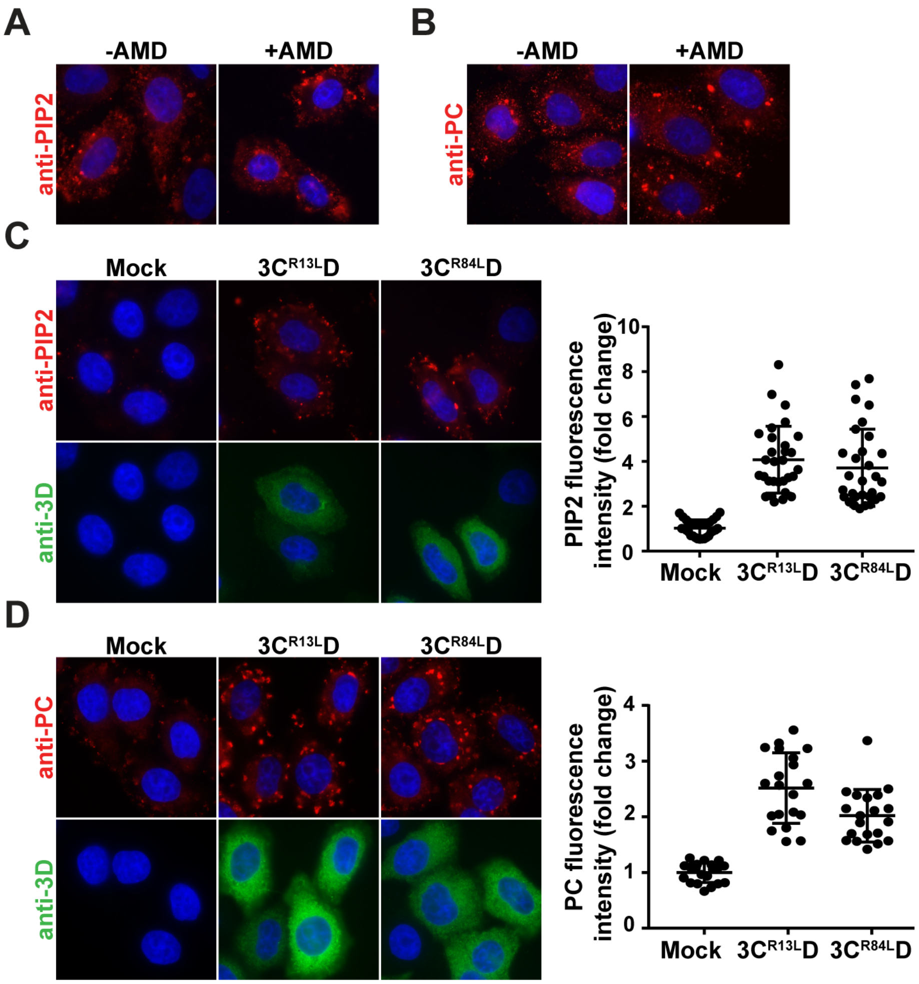

S5 Fig. **Induction of PIP2 and PC biosynthesis by 3CD is not sensitive to actinomycin D and does require induction of PI4P biosynthesis.** Effect of actinomycin D (AMD) on 3CD-mediated induction of PIP2 **(A)** and PC **(B)**. HeLa cells were maintained in the absence or presence of AMD (5 µg/mL) prior to transfection with 3CD mRNA. Cells were immunostained for PIP2 or PC (red) and 3CD (green); the nucleus was stained with DAPI (blue). The presence of AMD did not interfere with induction of PIP2 or PC. **(C)** The indicated 3CD derivatives were expressed individually in HeLa cells and immunostained for the presence of PIP2 (red) and 3CD (green). The nucleus was stained with DAPI (blue). Neither 3CD derivative impaired induction of PIP2. Quantification of PIP2 intensity per cell (n=30) was performed as described in the legend to **Fig. 1C**. The averages of the normalized values were: 1.03 ± 0.07 (SEM) in mock-transfected cells; 4.71 ± 0.27 (SEM) in $3C^{R13L}D$-transfected cells; 4.08 ± 0.27 (SEM) in $3C^{R84L}D$-transfected cells. The level of PIP2 induction observed in 3CD

mutant-transfected cells was not significantly different when compared to WT 3CD-transfected cells based on a Student's t-test. **(D)** The indicated 3CD derivatives were expressed individually in HeLa cells and immunostained for the presence of PC (red) and 3CD (green). The nucleus was stained with DAPI (blue). Neither 3CD derivative impaired induction of PC. Quantification of PC intensity per cell (n=20) was performed as described in the legend to **Fig. 1C**. The averages of the normalized values were: 1.00 ± 0.04 (SEM) in mock-transfected cells; 2.52 ± 0.14 (SEM) in $3C^{R13L}D$-transfected cells; 2.02 ± 0.11 (SEM) in $3C^{R84L}D$-transfected cells. The level of PC induction observed in 3CD mutant-transfected cells was not significantly different when compared to WT 3CD-transfected cells based on a Student's t-test.
