# Supplementary material for: Hijacking of multiple phospholipid biosynthetic pathways and induction of membrane biogenesis by a picornaviral 3CD protein
Source: PLoS Pathog. 2018 May 21;14(5):e1007086. doi: 10.1371/journal.ppat.1007086 (PMC5983871; doi:10.1371/journal.ppat.1007086)
Supplement: S1 Table — (PDF) [file ppat.1007086.s006.pdf]

S1 Table. **Oligonucleotides used in this study.**

Primer	Sequence	Description
1	5'-TTC GGG AAT TCG CCA CCA TGG GAC CAC TCC AGT ATA AAG AC-3'	Forward Primer to amplify 3AB
2	5'-CGT TAG CGG CCG CTT ACT ATT GTA CCT TTG CTG TCC GAA TG-3'	Reverse Primer to amplify 3AB
3	5'-TTC GGG AAT TCG CCA CCA TGG GAC CAG GGT TCG ATT AC-3'	Forward Primer to amplify 3CD
4	5'-CGT TAG CGG CCG CTT ACT AAA ATG AGT CAA GCC AAC-3'	Reverse Primer to amplify 3CD
5	5'-CGC AGT GGC TAT GGC TCT AAG AAA CAT TGT TAC AGC-3'	Forward primer to amplify 3C-K12L
6	5'-GCT GTA ACA ATG TTT CTT AGA GCC ATA GCC ACT GCG-3'	Reverse primer to amplify 3C-K12L
7	5'-CAG TGG CTA TGG CTA AAC TAA ACA TTG TTA CAG CAA C-3'	Forward Primer to amplify 3C-R13L
8	5'-GTT GCT GTA ACA ATG TTT AGT TTA GCC ATA GCC ACT G-3'	Reverse Primer to amplify 3C-R13L
9	5'-CTA AAG AGA AAT GAA AAG TTC CTA GAC ATT AGA CCA CAT ATA CC-3'	Forward Primer to amplify 3C-R84L
10	5'-GGT ATA TGT GGT CTA ATG TCT AGG AAC TTT TCA TTT CTC TTT AG-3'	Reverse Primer to amplify 3C-R84L
11	5'- GAA GGG TTG GAT AGT TAA CAT CAC CAG CCA GGT TC-3′	Forward primer for overlap PCR
12	5'- TCG CTT CAG GGC CGC GGC AAA CCC GTG TGA ACC G-3′	Reverse primer for overlap PCR
13	5'-ATT GGA AGA GCT TTA GAC CTC CCA GAG TAC TCA ACA TTG TAC GAT CGT TGG CTT GAC TCA-3'	Forward Primer to amplify 3D-L446D-R455D
14	5'-TGA GTC AAG CCA ACG ATC GTA CAA TGT TGA GTA CTC TGG GAG GTC TAA AGC TCT TCC AAT-3'	Reverse Primer to amplify 3D-L446D-R455D
15	5'-TTC GGG AAT TCG CCA CCA TGG GAC CAC TCC AGT ATA AAG AC-3'	Forward Primer to amplify 3A
16	5'-CGT TAG CGG CCG CTT ACT ACT GGT GTC CAG CAA ACA GTT TAT AC-3'	Reverse Primer to amplify 3A
