# Supplementary material for: Hijacking of multiple phospholipid biosynthetic pathways and induction of membrane biogenesis by a picornaviral 3CD protein
Source: PLoS Pathog. 2018 May 21;14(5):e1007086. doi: 10.1371/journal.ppat.1007086 (PMC5983871; doi:10.1371/journal.ppat.1007086)
Supplement: S2 Table — (PDF) [file ppat.1007086.s007.pdf]

S2 Table. **Statistical analysis of all experimental data.**

Fig No.	Samples	Mean ± SEM	p-value	n
Fig 1	Mock	1.00 ± 0.02		30
	3AB	0.81 ± 0.04		27
	3CD	4.98 ± 0.43		30
	3A	0.53 ± 0.03		35
	Mock vs. 3AB		<0.0001	
	Mock vs. 3CD		<0.0001	
	Mock vs. 3A		<0.0001	
	3AB vs. 3CD		<0.0001	
	3AB vs. 3A		<0.0001	
	3CD vs. 3A		<0.0001	
Fig 2	3CD -PIK93	$1.84e^{8} \pm 1.19e^{7}$		25
	3CD +PIK93	$7.19e^{7} \pm 5.18e^{6}$		25
	3CD -PIK93 vs. WT+PIK93		<0.0001	
Fig 3	Mock	0.43 ± 0.03		3
	3CD	1.77 ± 0.03		3
	Mock vs. 3CD		<0.0001	
Fig 6 (B)	Mock	1.00 ± 0.04		30
	$3CD^{m}$	1.06 ± 0.04		30
	$3C^{m}D$	1.10 ± 0.04		30
	$3CD^{m}$+$3C^{m}D$	3.08 ± 0.13		30
	Mock vs. $3CD^{m}$		0.2602	
	Mock vs. $3C^{m}D$		0.089	
	Mock vs. $3CD^{m}$+ $3C^{m}D$		<0.0001	
	$3CD^{m}$ vs. $3C^{m}D$		0.5574	
	$3CD^{m}$ vs. $3CD^{m}$+ $3C^{m}D$		< 0.0001	
	$3C^{m}D$ vs. $3CD^{m}$+ $3C^{m}D$		< 0.0001	
Fig 6 (D)	Mock	0.42 ± 0.02		3

	$3CD^{m}$	0.37 ± 0.07		3
	$3C^{m}D$	1.27 ± 0.09		3
	Mock vs. $3CD^{m}$		0.4498	
	Mock vs. $3C^{m}D$		0.0007	
	$3CD^{m}$ vs. $3C^{m}D$		0.0012	
Fig 7 (B)	Mock	1.00 ± 0.03		42
	3CD	2.60 ± 0.13		30
	3CD+3AB	2.47 ± 0.10		30
	3CD+3A	0.83 ± 0.03		35
	Mock vs 3CD		<0.0001	
	Mock vs 3CD+3AB		<0.0001	
	Mock vs 3CD+3A		<0.0001	
	3CD vs 3CD+3AB		0.1490	
	3CD vs 3CD+3A		<0.0001	
	3CD+3AB vs 3CD+3A		<0.0001	
Fig 8 (B)	3CD	170 ± 10		3
	$3CD^{m}$	330 ± 30		3
	$3C^{m}D$	80 ± 10		3
	3CD vs $3CD^{m}$		0.0072	
	3CD vs $3C^{m}D$		0.0031	
	$3CD^{m}$ vs $3C^{m}D$		0.0014	
Fig 9 (A)	Mock	1.00 ± 0.05		30
	Infection	6.62 ± 0.37		30
	Mock vs. Infection		<0.0001	
Fig 9 (B)	Mock	1.00 ± 0.04		30
	Infection	3.03 ± 0.15		30
	Mock vs. Infection		<0.0001	
Fig 9 (C)	Mock	1.00 ± 0.07		30
	3CD	4.37 ± 0.25		30

	$3CD^m$	3.21 ± 0.16		30
	$3C^mD$	3.82 ± 0.25		30
	Mock vs. 3CD		<0.0001	
	Mock vs. $3CD^m$		<0.0001	
	Mock vs. $3C^mD$		<0.0001	
	3CD vs. $3CD^m$		0.0002	
	3CD vs. $3C^mD$		0.1264	
	$3CD^m$ vs. $3C^mD$		0.0447	
Fig 9 (D)	Mock	1.00 ± 0.02		45
	3CD	2.18 ± 0.07		45
	$3CD^m$	1.89 ± 0.07		45
	$3C^mD$	2.06 ± 0.10		28
	Mock vs. 3CD		<0.0001	
	Mock vs. $3CD^m$		<0.0001	
	Mock vs. $3C^mD$		<0.0001	
	3CD vs. $3CD^m$		0.0041	
	3CD vs. $3C^mD$		0.3129	
	$3CD^m$ vs. $3C^mD$		0.1583	
S1 Fig	Mock	1.28 ± 0.14		4
	Infection	8.38 ± 0.81		4
	Mock vs. Infection		0.0001	
S3 (B) Fig	Mock	1.06 ± 0.02		20
	$3C^{R13L}D$	1.10 ± 0.06		20
	$3C^{R84L}D$	0.96 ± 0.05		20
	Mock vs. $3C^{R13L}D$		0.5386	
	Mock vs. $3C^{R84L}D$		0.0756	
	$3C^{R13L}D$ vs. $3C^{R84L}D$		0.0798	
S3 (D) Fig	Mock	0.43 ± 0.03		3
	$3C^{R13L}D$	1.23 ± 0.18		3

	$3C^{R84L}D$	1.50 ± 0.15		3
	Mock vs. $3C^{R13L}D$		0.0112	
	Mock vs. $3C^{R84L}D$		0.0024	
	$3C^{R13L}D$ vs. $3C^{R84L}D$		0.3169	
S5 (C) Fig	Mock	1.03 ± 0.07		30
	3CD	4.71 ± 0.27		30
	$3C^{R13L}D$	4.08 ± 0.27		30
	$3C^{R84L}D$	3.71 ± 0.31		30
	Mock vs. 3CD		<0.0001	
	Mock vs. $3C^{R13L}D$		<0.0001	
	Mock vs. $3C^{R84L}D$		<0.0001	
	3CD vs. $3C^{R13L}D$		0.1053	
	3CD vs. $3C^{R84L}D$		0.0194	
	$3C^{R13L}D$ vs. $3C^{R84L}D$		0.3827	
S5 (D) Fig	Mock	1.00 ± 0.04		20
	3CD	2.39 ± 0.15		20
	$3C^{R13L}D$	2.52 ± 0.14		20
	$3C^{R84L}D$	2.02 ± 0.11		20
	Mock vs. 3CD		<0.0001	
	Mock vs. $3C^{R13L}D$		<0.0001	
	Mock vs. $3C^{R84L}D$		<0.0001	
	3CD vs. $3C^{R13L}D$		0.5371	
	3CD vs. $3C^{R84L}D$		0.0523	
	$3C^{R13L}D$ vs. $3C^{R84L}D$		0.0078	
